# Quantification of brain-wide vascular resistivity via ultrafast Doppler in human neonates helps early detection of white matter injury

**DOI:** 10.1177/0271678X241232197

**Published:** 2024-02-10

**Authors:** Flora Faure, Jérôme Baranger, Marianne Alison, Béatrice Boutillier, Alice Frérot, Chung Lim, Grégory Planchette, Mickael Prigent, Mickaël Tanter, Olivier Baud, Valérie Biran, Charlie Demené

**Affiliations:** 1 Physics for Medicine, INSERM U1273, CNRS, ESPCI, PSL Research University, Paris, France; 2 Department of Radiology, Assistance Publique-Hôpitaux de Paris, Robert Debré Children’s Hospital, University Paris Cité, Paris, France; 3 Neonatal Intensive Care Unit, Assistance Publique-Hôpitaux de Paris, Robert Debré Children’s Hospital, University Paris Cité, Paris, France; 4 Department of Pediatric, University Hospital of Geneva, University of Geneva, Geneva, Switzerland. O.B. is also with INSERM U1141, Robert Debré Children’s Hospital, University Paris Cité, Paris, France; 5 I2D2, INSERM U1141, University Paris Cité Paris, France

**Keywords:** Brain, neonates, ultrafast Doppler, vascular resistivity, white matter injury

## Abstract

Preterm birth is associated with cerebrovascular development disruption and can induce white matter injuries (WMI). Transfontanellar ultrasound Doppler is the most widely used clinical imaging technique to monitor neonatal cerebral vascularisation and haemodynamics based on vascular indexes such as the resistivity index (RI); however, it has poor predictive value for brain damage. Indeed, these RI measurements are currently limited to large vessels, leading to a very limited probing of the brain’s vascularisation, which may hinder prognosis. Here we show that ultrafast Doppler imaging (UfD) enables simultaneous quantification, in the whole field of view, of the local RI and vessel diameter, even in small vessels. Combining both pieces of information, we defined two new comprehensive resistivity parameters of the vascular trees. First, we showed that our technique is more sensitive in the early characterisation of the RI modifications between term and preterm neonates and for the first time we could show that the RI depends both on the vessel diameter and vascular territory. We then showed that our parameters can be used for early prediction of WMI. Our results demonstrate the potential of UfD to provide new biomarkers and pave the way for continuous monitoring of neonatal brain resistivity.

## Introduction

Perinatal brain injuries result from a complex combination of defects in perfusion and cerebral inflammation. In particular, the most common cerebral morbidities in preterm neonates (born before 37 weeks of gestation), intraventricular haemorrhage (IVH) and diffuse periventricular leukomalacia (PVL),^1,2^ have been associated with impaired cerebral blood flow regulation.^3^ These conditions often lead to poor neurodevelopmental outcomes^4^ and lifelong disabilities such as cerebral palsy, dyspraxia (also known as developmental coordination disorder) or attention deficit disorder.

Systemic measurements such as peripheral arterial blood pressure generally correlate quite poorly with both brain perfusion and neurodevelopmental outcomes. Hence, the benefit of treating peripheral hypotension to improve cerebral perfusion remains unclear.^5^ This might be related to the poor maturation of cerebral autoregulation^6^ in the most premature neonates, which disrupts the expected transfer function between cardiac output and cerebral circulation.^7^ Therefore, measuring haemodynamics directly in the brain may be helpful to monitor the true brain status of these neonates. Two aspects can be stressed here: first, monitoring smaller vessels compared with the current clinical practice may help to obtain information that has better prognostic value, because PVL development usually starts from the end zones of vessels.^3^ Second, having a quantitative biomarker that reflects the general haemodynamic status of the vascular tree at multiple scales (and in particular the smaller ones) might help to efficiently predict the neurodevelopmental outcome. Such a biomarker has been sought in perfusion imaging. Many techniques have been reported to image brain perfusion in neonates,^8^ but they are invasive (SPECt, PET and CT) or difficult to apply (MRI) in the neonatal intensive care unit (NICU). Although non-invasive and bedside near-infrared spectroscopy (NIRS) seems promising to monitor cerebral blood volume and oxygenation, it remains superficial, overlooks deep structures and its clinical adoption remains elusive.^9^ To date, transfontanellar ultrasound Doppler imaging remains the most widely used technique in the NICU for neonatal brain cerebrovascular monitoring.

Currently, there are two ‘conventional’ complementary ultrasound Doppler imaging modes: colour Doppler and pulse wave Doppler (PWD). In colour Doppler, the medium is scanned line by line, acquiring a few time samples per line, resulting in a dynamic map of the estimated blood flow velocity in all large vessels within the field of view (FOV). This technique has relatively poor sensitivity and low precision regarding the blood flow velocity measurements. On the other hand, PWD is used to acquire sensitive and quantitative temporal information about blood flow velocities but can be performed in only one region of interest (ROI) of the FOV by focusing the ultrasound in this location. Hence, there is disjuncture between imaging and quantification, with the latter being possible in only a set of manually chosen locations, and not in the entire FOV. This quantification often relies on the resistivity index (RI), also called the Pourcelot index, which reflects the resistance to blood flow created by the microvascular bed surrounding the point of measurement. It was defined in 1972 by Léandre Pourcelot as

(1) $$\mathrm{RI}=\frac{\mathrm{PSV}-\mathrm{EDV}}{\mathrm{PSV}}$$

where peak systolic velocity (PSV) and end-diastolic velocity (EDV) are measured on the PWD spectrum. While the RI has been shown to reflect certain aspects of cerebral blood circulation, its relevance for monitoring the cerebral status in neonates has not been clearly established. This might be due to several interlinked reasons: (1) the RI is generally measured in stereotyped arterial locations, such as the A3 segment of the anterior cerebral artery (ACA) (next to the genu of the corpus callosum), whose local anatomy and vessel diameter may change across individuals, increasing the intra-group variances; (2) those stereotyped locations may poorly reflect the vascular resistivity status in other parts of the total brain vascular structure; (3) the poor sensitivity of conventional Doppler prevents measurement of the RI in small vessels and downstream in the vascular tree; and (4) RI measurements are performed only during short periods spaced over time,^10^ as they need to be done by a trained operator, preventing a finely sampled and long-lasting monitoring of the cerebrovascular status. To sum up, resistivity measurements give very fragmented snapshots of cerebral circulation in both space and time.

The development of ultrafast ultrasound – and in particular ultrafast Doppler (UfD), which uses plane-wave compounding instead of conventional focused beams scanning^11^ – enables a 50-fold gain in sensitivity and greater robustness to motion artefacts.^12^ Besides its increased sensitivity, UfD bridges the gap between imaging and quantification found in conventional Doppler imaging modes. The utilisation of plane waves enables the collection of numerous simultaneous samples throughout the entire FOV. Hence, one can simultaneously gather quantitative temporal information about blood flow velocity in all vessels, even small ones.^13^ It is then possible to study the cerebrovascular RI in the whole imaging plane with only one acquisition, as has been shown in previous studies with RI mapping in neonatal brains,^14^ evaluation of blood flow velocities in small vessels of the basal ganglia^15^ and monitoring of brain resistivity changes in a cardiac resuscitation model.^16^ Comparable values of brain hemodynamic parameters (PSV, EDV and the RI) in neonates are obtained with UfD and conventional Doppler ultrasound.^17^

In this article we show that by using UfD, we can derive automated RI measurements over the whole brain and the local vessel diameter, enabling for the first time the ability to relate the two quantities throughout the vascular tree. We present a quantitative assessment of cerebrovascular RI in generally overlooked small vessels and quantification of the RI versus vessel diameter in a large cohort of human neonates. In our cohort, this RI versus diameter modelling at 21 days of life was predictive of the occurrence of white matter lesions at term-equivalent age (TEA; 40 weeks). Our results demonstrate the potential of UfD and brain-wide resistivity quantification as new biomarkers and pave the way for UfD to be used for continuous monitoring of neonatal brain resistivity by removing the need for a trained operator to pinpoint the measurement locations.

## Methods

### Patient recruitment

**Figure 1.**
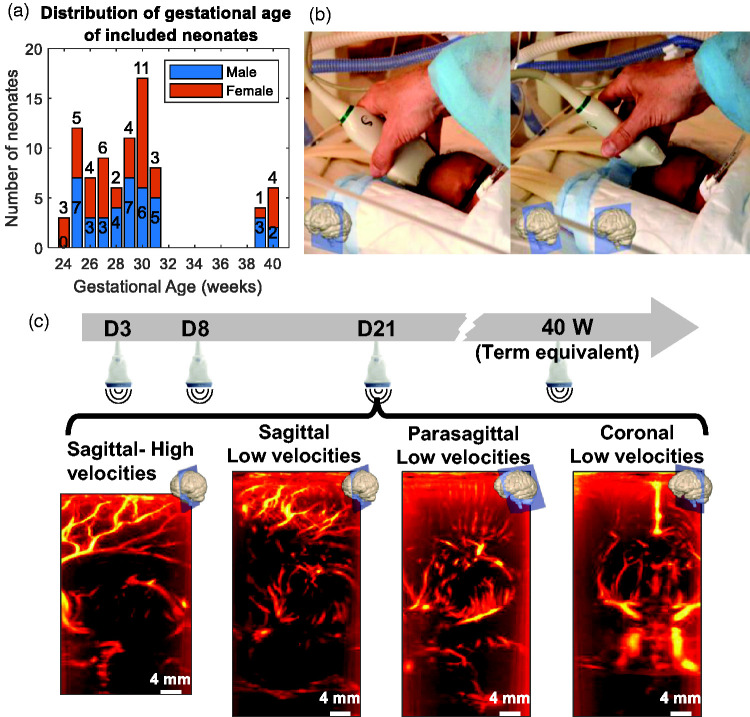
Age distribution and ultrafast Doppler imaging protocol for neonates included in the study. (a) Headcount of the neonates included in the study according to gestational age at birth (in weeks) and sex (blue = male, orange = female) and (b) Positioning of the probe by a trained operator in the coronal (left) and sagittal (right) planes. (c) Timeline of acquisitions for a preterm patient: at each time point (postnatal day 3 [D3], D8, D21 and at term-equivalent age [TEA, meaning 40 weeks]), four different acquisitions were performed with UfD using either a sequence dedicated to the imaging of high blood velocities (3200 Hz frame rate) or one dedicated to low blood velocities (1600 Hz) and either a sagittal or coronal plane.

We recruited a total of 84 neonates born at Robert Debré University Hospital, Paris, France, after obtaining written consent from the parents. The cohort included 74 very preterm neonates born between 24^0/7^ and 31^6/7 ^weeks of gestation, with a mean gestational age of 28.5 ± 2.2 weeks, and 10 control full-term neonates born between 39^0/7^ and 40^6/7 ^weeks of gestational age, with a mean gestational age of 40.1 ± 0.8 weeks (see Figure 1(a) for additional details about the headcount, gestational age and sex). Additional patient information and prenatal history are provided in Supplemental Table 1. We registered this study at EudraCT (ID: 2012-A01530-43) and ClinicalTrials.gov (ID: NCT02042716).

The protocol was approved by the local ethical committee (Robert Debré Hospital, Comité de Protection des Personnes #120601, BELUGA protocol, promoted by INSERM, Institut National de la Santé et de la Recherche Médicale, French Health Institute) and strictly complied with the ethical principles for medical research involving human subjects of the World Medical Association Declaration of Helsinki.

### UfD data acquisition

We acquired transfontanellar UfD images at the bedside in the sagittal and coronal planes by using a 6 MHz linear ultrasound probe (SL10-2 Supersonic Imagine, 192 elements, pitch 0.2 mm; Figure 1(b)). A trained operator manually placed the probe over the anterior fontanel using both B-Mode imaging and vascular landmarks in colour Doppler to select the desired planes.

We programmed two different ultrasound sequences (see the characteristics in Table 1) on an Aixplorer ultrasound system, favouring either the non-aliased sampling of high velocities (up to 40 cm/s) or sensitivity to small vessels (such as lentriculostriate arteries), via the use of a high (3200 Hz) compounded frame rate or a larger number (5) of compounded angles, respectively. The FOV is a window with a width of 25 mm (using the 128 central elements) and a depth of up to 60 mm. The acquisition time for both sequences, although short (<1 s), lasted long enough to cover 2–3 cardiac cycles. The calibrated acoustic outputs strictly complied with the Food and Drug Administration Track 3 recommendations (MI < 1.9, ISPTA < 720 mW/cm^2^ and ISPPA < 190 W/cm^2^, see Table 1).

**Table 1.** Characteristics of the ultrasound sequences.

	High-velocity ultrasound sequence	Low-velocity ultrasound sequence
Depth (mm)	[4 50]	[2 60]
Pulse repetition frequency (PRF) (Hz)	9600	8000
Angle for tilted plane waves	(−3°, 0,3°)	(−5°, −3°, −1°, 1°, 3°)
Frame rate (Hz)	3200	1600
Duration of acquisition (s)	0.78	1
Number of compounded ultrasound frames	2500	1600
Mechanical Index (MI)	0.5	0.5
ISPTA (mW/cm²)	190	158
ISPPA (W/cm²)	62	62

ISPTA: intensity spatial-peak temporal average; ISPPA: intensity spatial-peak pulse average.

We performed acquisitions for preterm neonates on postnatal day 3 (D3), D8, D21 and at TEA (40 weeks). We only performed one acquisition for term neonates – at D3, corresponding to TEA. We obtained a total of four acquisitions in different planes with both sequences for each patient at each day of measurement (Figure 1(c)). Missing data, which represent 14% of all acquisitions, were due to hospital leave or transfer to another hospital before TEA.

### High-sensitivity power doppler processing

We filtered beamformed and compounded ultrasound data by using a singular value decomposition (SVD) clutter filter^18^ to retain only components from blood and to remove the surrounding tissue contribution. We used adaptive thresholding based on the projection of the pixels of the similarity matrix of the spatial singular vectors on the main diagonal^19,20^ to achieve optimal blood filtering with a fast-computing time. Integrating the energy of the filtered signal for each voxel over the time of acquisition of the block gives a power Doppler image where the intensity is proportional to the number of moving red blood cells in the voxel.^21^

### Automated local vessel diameter estimation

**Figure 2.**
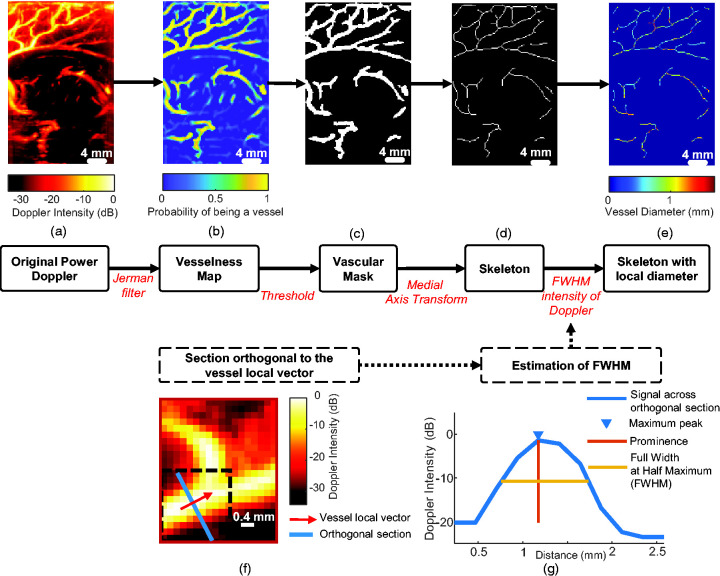
Processing pipeline to determine the local vessel diameter along the vascular tree. (a) Original Power Doppler (PWD) image. (b) A vesselness map (i.e the map of probability for a pixel to belong to a vessel) is obtained by applying a vascular enhancement filter (Jerman Filter) (c) A vascular mask is obtained by thresholding the vesselness map. (d) The skeleton of the vascular mask is extracted by using medial axis transform. (e) The skeleton of the vascular tree shows mapping of the local diameter at each point is obtained after full width at half maximum (FWHM) estimation (see (f) and (g) for details for a given pixel of the skeleton). (f) The local orientation vector of the vessel is determined for a pixel of the skeleton (red arrow) and the section orthogonal to the orientation vector is also defined (blue line) and (g) The PWD intensity along the orthogonal direction is plotted in blue and the diameter is estimated from the FWHM plotted in yellow.

From the power Doppler image (Figure 2(a)), we computed a vesselness map (Figure 2(b)) by using a vascular enhancement filter (MATLAB code vesselness2D implementing the Jerman filter^22^ available at https://github.com/timjerman/JermanEnhancementFilter). This Hessian-based filter algorithm leverages the assumption that a vessel can be modelled by a tube of radius R with a Gaussian distribution of the intensity of blood flow in a plane orthogonal to the tube. In this model, the maximum blood flow intensity lies in the middle of the vessel and its standard deviation corresponds to the radius. At the end, it gives a vesselness map that represents the probability for each pixel to belong to a vessel.

Next, we thresholded the vesselness map to obtain a binary vascular mask (Figure 2(c)) from which we selected the centreline pixels, also called the skeleton (Figure 2(d)). The skeleton is the locus of the centres of the maximum inscribed circle among the circles that touch the boundary more than once. We obtained it by using the MATLAB-based ‘bwskel’ skeletonisation algorithm, relying on medial axis transform (MAT) with a connectivity of 4.

The last step was to retrieve, for each pixel of the skeleton, the local diameter defined as the full width at half maximum (FWHM) of the intensity of the power Doppler image. As the diameter is likely to be overestimated at intersections by the FWHM method, we removed from the skeleton the pixels located at the intersection and their neighbours on a disk of radius 4 (referred to as “Masked skeleton” in the figures). We first defined a local orientation vector for each point of the skeleton to evaluate the local angle θ it forms with a vertical vector (Figure 2(f)). We used this angle θ to rotate an 11 × 11 neighbourhood of the Doppler image centred on the skeleton pixel to position the vessel vertically (image rotation is a means to perform the transverse interpolation efficiently). Then we evaluated the FWHM on the intensity of the orthogonal midline (Figure 2(g)) by using the MATLAB function ‘findpeaks’.

After performing the aforementioned steps, we obtained a map of the local diameter for every pixel within the skeleton (Figure 2(e)). We validated the automated diameter extraction by using a Doppler phantom with several canals of a small diameter.

### Spectral analysis of UfD data and calculation of the local RI

**Figure 3.**
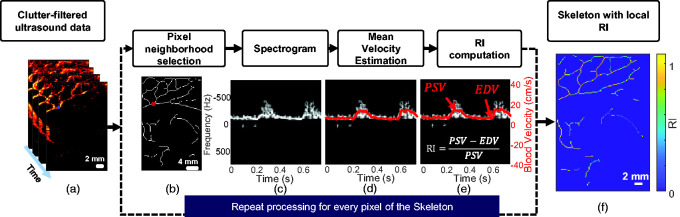
Processing pipeline to calculate local resistivity along the vascular tree. (a) A stack of ultrafast Doppler (UfD) ultrasound data after singular value decomposition (SVD) clutter-filtering. (b) Selection of a 5 × 5 neighbourhood around a pixel of interest (c) Averaged spectrogram of the previously selected area. A spectrogram is obtained for each pixel by combining the power spectrum of the fast Fourier transform on multiple time windows and then by averaging (in magnitude) the spectrograms. (d) Mean Doppler frequency estimation (red line), which is proportional to blood velocity. (e) Calculation of the resistivity index (RI) based on the peak systolic velocity (PSV) and end-diastolic velocity (EDV) and (f) The skeleton of the vascular tree shows mapping of the local resistivity at each point.

The spectral content of the cluttered-filtered ultrasound data (Figure 3(a)) is directly influenced by the speed of local ultrasound scatterers, as the Doppler frequency $f_{\mathrm{Doppler}}$ is directly linked to the velocity V of the moving red blood cells according to

(2) $$V=\frac{f_{\mathrm{Doppler}}{\;c}_{0}}{{2f}_{\mathrm{Pulse}}cos(\theta )}$$

where $c_{0}$ is the speed of sound, $f_{\mathrm{Pulse}}$ is the probe frequency and θ is the angle between the wave vector of the incoming ultrasound wave and the velocity vector of the moving scatterer. We must emphasise that this spectrum is broadened by the speed distribution of the many moving red blood cells within the voxel, the diversity of the ultrasound wave vectors (due to the plane wave compounding) and the diversity of the angles in reception (beamforming with a large aperture). See previous publications for additional details.^23,24^

To obtain blood velocity along the cardiac cycle, we performed short-time Fourier transform of the clutter-filtered ultrasound data by using 40 ms sliding-windows (with 37 ms of overlap) of the signal in every pixel. The spectrogram (i.e. mapping the frequency intensity over time) is the magnitude of the short-time Fourier transform (Figure 3(c)). Then, the mean Doppler frequency can be estimated from this spectrogram as described by Demené et al.,^14^ and mean blood flow velocity (red line in Figure 3(d)) can be calculated by using equation (2). From the mean blood flow velocity over time, we extracted PSV and EDV to calculate the RI using equation (1) (Figure 3(e)). Note that the ratio cancels out the cos(θ) correction, making this measurement angle independent. UfD enables performing this task simultaneously for every pixel of the FOV, and therefore of the skeleton (Figure 3(f)).

We applied several steps to make the PSV and EDV determination for a given pixel less sensitive to noise. First, we averaged the magnitude of the spectrograms over a 5-pixel neighbourhood (Figure 3(b)). Then, we set the pixels of the spectrogram whose intensities were <10% of the maximum intensity – which most likely represent noise – to zero. Finally, for each acquisition, we calculated an average spectrogram over the entire image to determine the averaged time points of PSV and EDV. Then we restricted the determination of the precise time points of PSV and EDV for each pixel of the skeleton to a time window of 12.5 ms, centred on their respective averaged time points.

After spectrogram computation, an aliasing phenomenon can sometimes be observed due to inadequate sampling of high blood flow velocity in large vessels. Thus, we implemented a de-aliasing step, which has been validated and published by Demené et al.^14^ It is based on the reasonable assumption that in a vessel the blood flows in one direction only, either up or down, and thus enables one to double the maximum flow speed that can be measured with ultrasound imaging for a given sampling rate.

### Discrimination between venous and arterial circulation

At this stage of the process, we calculated the RI in all vessels without discrimination between veins and arteries. Because the flow is not pulsatile in veins, the RI is generally low and its fluctuations are quite sensitive to noise. Hence, we wanted to keep only the RI from arteries. We evaluated two ROIs in this study: one including the branches of the ACA (ACA branches ROI) and one including the thalamic arteries (thalamic ROI). By examining the histogram of values of the RI from all acquisitions of all patients for each ROI, we could distinguish two Gaussian distributions with different means. We set the threshold to separate veins from arteries as the intersection between these two Gaussians distributions by applying a two-component Gaussian mixture model (GMM) to the histogram. It provided a threshold of 0.357 for the ACA branches and 0.225 for the thalamic vessels.

### Resistivity versus local diameter analysis

**Figure 4.**
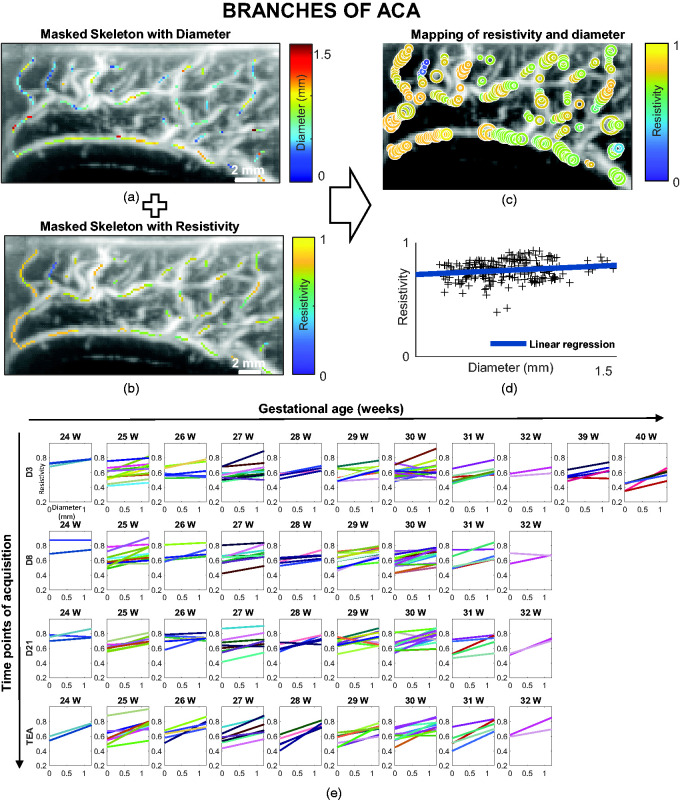
Resistivity versus artery diameter analysis on the defined anterior cerebral artery (ACA) branches region of interest (ROI). (a) Doppler image (in greyscale) with the local diameter (in the given colour scale) superimposed in ACA branches ROI. (b) Doppler image (in greyscale) with the local resistivity (in the given colour scale) superimposed in ACA branches ROI. (c) Coincident mapping of resistivity and diameter (the size of the circles is proportional to the diameter and the colours represent resistivity according to the colour map). (d) The resistivity index (RI) and diameters can also be gathered in a resistivity versus diameter plot (black crosses), before performing linear regression of resistivity as a function of diameter (blue line) and (e) RI versus diameter linear regression for all patients grouped by gestational age at birth (in weeks [W]) and day of acquisition (postnatal day 3 [D3], D8, D21 and term-equivalent age [TEA]) in ACA branches. Each patient is represented by a specific colour.

For a given UfD acquisition, by combining information about the local diameter of arteries (Figure 4(a)) and the local value of resistivity (Figure 4(b)) for each pixel of the skeleton, we could calculate a map with resistivity and diameter values for our studied ROIs (Figure 4(c)). We selected the ACA branches ROI based on UfD acquisitions with a high-velocity ultrasound sequence (Table 1) and the thalamic ROI based on UfD acquisitions with a low-velocity ultrasound sequence (Table 1). For quantification, we gathered the measurement for each ROI in a resistivity versus diameter plot and performed a linear regression (Figure 4(d)). We performed linear regression between the RI and the diameter for each patient and time point of the protocol (D3, D8, D21 and TEA) in the ACA branches (Figure 4(e)) and thalamic ROIs (Supplemental Figure 1).

We focused on two parameters of interest: the slope of the linear regression, which reflects the evolution of resistivity along the vascular tree, and the value of that linear model for a diameter of 0.2 mm. Because our spatial resolution limit is 0.2 mm, we considered the value of the regression for a diameter of 0.2 mm to be the average limit of the RI in the smallest vessels; we call it the ‘**RI in small vessels’**.

Then, we determined the average of the linear regressions of each group formed according to the gestational age (preterm/term), the day of postnatal acquisition (D3/D8/D21/TEA) and the ROIs (ACA branches/thalamus). For each group, the mean linear regression $\bar{y}$ is defined as

(3) $$\bar{y}=\bar{m}x+\bar{c}$$

where $\bar{m}$ is the mean of the slopes of the group, $\bar{c}$ is the mean of intercepts of the group and x is the vector of all values of diameter. The 95% confidence interval (CI) can be estimated with the formula

(4) $$CI=\bar{y}\pm 1.96\sigma_{y}\times \sqrt{\frac{\frac{1}{n}+{\left(x-\bar{x}\right)}^{2}}{\sum {\left(x_{i}-\bar{x}\right)}^{2}}}$$

where $\sigma_{y}$ is the standard deviation of the values of all linear regressions at the abscissa $\bar{x}$ (mean of *x*) and *n* is the number of linear regressions (i.e. the number of patients in the group).

### Statistical analysis

We separated the cohort into two groups for comparison: preterm neonates born between 24 and 32 weeks of gestation and term neonates born between 39 and 40 weeks of gestation. Before performing the statistical analysis, we evaluated the normality of the data by using the Shapiro–Wilk test. We used parametric tests for normally distributed data and non-parametric tests for non-normally distributed data. The results are presented as the median [25th percentile 75th percentile] when they were not normally distributed and as the mean ± standard deviation when they were normally distributed.

We compared our measurements in the ACA branches (RI in small vessels and slopes) between the day of acquisition for preterm neonates (D3, D8, D21 and TEA) to the control group of term neonates at D3 with one-way analysis of variance (ANOVA) followed by the post hoc Bonferroni correction for multiple comparisons. We compared the RI in the A3 segment of the ACA with the non-parametric Kruskal–Wallis test, as the data were not normally distributed, followed by the post hoc Bonferroni correction for multiple comparisons.

We compared the slopes between the ROIs (ACA branches and thalamic arteries) for all groups and days of acquisition by using a two-sample t-test. We compared the RI in small vessels of all groups and days of acquisition by using the Wilcoxon signed-rank test, as the data in the thalamic arteries were not normally distributed.

We used a two-sample t-test to compare the slopes of the RI between patients with pathological and normal MRI.

### MRI data acquisition and classification

In the context of neonatal care, all preterm neonates with a gestational age of <28 weeks at birth underwent MRI examinations at TEA. Premature infants with a gestational age >28 weeks may also have MRI scans based on specific needs. All examinations were performed on a 1.5 T Medical System with standard head coil, without sedation and during the post-feeding period. The following sequences were used:

- an axial and sagittal T2-weighted single-shot turbo spin-echo sequence (repetition time (TR) 16900 ms/echo time (TE) 100 ms, NEX 1, matrix 256 × 196, FOV 230 × 194 mm, section thickness 3 mm);
- an axial T2-weighted dual turbo spin-echo sequence (TR 3000 ms, TE 8.8/110 ms, matrix 256 × 256, FOV 230 × 172 mm, section thickness 4 mm, gap 1 mm);
- a coronal T2-weighted turbo spin-echo (TR 4765 ms, TE 100 ms, matrix 256 × 152, FOV 200 × 159 mm, section thickness 2 mm, no gap);
- an axial and sagittal T1-weighted turbo spin-echo sequence (TR 560 ms, TE 12 ms, matrix 212 × 117, FOV 190 × 132 mm, section thickness 3 mm, gap 0.3 mm);
- an axial T2 fast field echo-planar imaging sequence (TR 2190 ms, TE 44 ms, matrix 212 × 139, FOV 190 × 170 mm, section thickness 4 mm, gap 1 mm).

Based on abnormalities of the morphology, the volume or the signal, we classified MRI images of the preterm neonates as pathological or normal.

## Results

### Validation of the diameter estimation

We use a Doppler phantom with three tubes of known diameter (0.46, 0.86 and 1.60 mm) to test our estimation of the diameter. These diameters are in the range of the typical vessel diameters observed during our study. We tested the algorithm and found consistent values on the power Doppler images of the Doppler phantom. The average diameter estimate for pixels of the skeleton was 0.52 ± 0.28 mm for a true value of 0.46 mm, 0.75 ± 0.19 mm for a true value of 0.86 mm and 1.34 ± 0.11 mm for a true value of 1.60 mm.

### The RI increases with diameter within a vascular tree

Resistivity versus artery diameter linear regressions are presented for each patient and day of acquisition in Figure 4(e) for the ACA branches ROI and in Supplemental Figure 1 for the thalamic ROI. For the regression of RI versus diameter, we noted that for most patients the slope was positive, that is, the RI increases as the diameter of the vessel increases. On average, the RI versus diameter slope was 0.08 ± 0.07 mm^−1^ for the ACA branches ROI (significantly > 0; *p* *< *0.001) and 0.03 ± 0.08 mm^−1^ for the thalamic arteries (significantly > 0; *p < *0.001). Ecury-Goosen and coauthors described higher resistivity in large vessels compared with small vessels.^25^ However, as the diameter cannot be quantified with conventional ultrasound, their assumption of size was made by relying on anatomical preconceptions and by comparing arteries belonging to different vascular trees (e.g. thalamic arteries are considered smaller than the ACA and have lower resistivity). We show here that the picture is more complex, as both vessel diameter and anatomical location have an influence on the resistivity.

### From D3, the RI in small vessels is higher in preterm than term neonates

**Figure 5.**
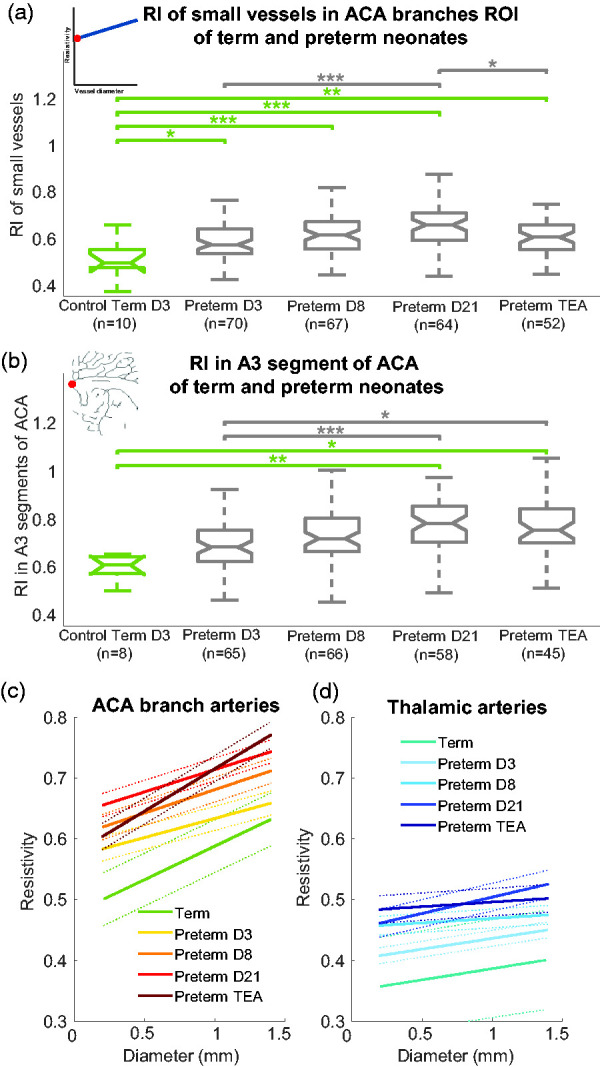
Comparison of resistivity for term and preterm neonates according to postnatal days. For (a) and (b), notches on the boxplots represents the 95% confidence interval for the median, calculated as $\mathrm{median}\pm 1.57\times \frac{\mathrm{IQR}}{\sqrt{n}}$ , where IQR is the interquartile range and n is the number of observations. (a) RI in small vessels in the ACA branches evaluated by the value of regression for a diameter of 0.2 mm, (red dot on the linear regression) grouped by day of acquisition (postnatal day 3 [D3], D8, D21 and term-equivalent age [TEA]) and the gestational age group. One-way analysis of variance was performed followed by post hoc Bonferroni correction for multiple comparisons (*p < 0.05, **p < 0.01, ***p < 0.001) (b) Resistivity measured in the A3 segments of the ACA, next to the genu of the corpus callosum grouped by day of acquisition (D3, D8, D21 and TEA) and by the gestational age group. As the data were not normally distributed, the Kruskal–Wallis test was performed followed by post hoc Bonferroni correction for multiple comparisons (*p < 0.05, **p < 0.01, ***p < 0.001). (c–d) Average linear regression of all patients per group between the RI and the diameter for term and preterm neonates at each time point of the protocol (D3, D8, D21, TEA) (solid lines) in the ACA branches (c) and thalamic arteries (d). The 95% confidence bounds are represented with dashed lines (see methods).

We compared the RI in small vessels from the ACA branches ROI between preterm and term at each day of acquisition. We found that as early as D3, the RI in small vessels in preterm neonates (0.58 ± 0.08) was higher compared with full-term neonates (0.50 ± 0.08) (*p < *0.05) and remained higher at D8, D21 and TEA (*p < *0.001, *p < *0.001 and *p < *0.01, respectively) (Figure 5(a), green significance stars). We also carried out this comparison by separating the premature neonates into two subgroups: those born between 24 and 28 weeks and those born between 28 and 32 weeks; however, there were no significant differences between these subgroups (Supplemental Figure 2).

When assessing this evolution of resistivity in the preterm neonates based on the usual RI measurement in the ACA next to the genu of the corpus callosum (A3 segment of the ACA), the difference in the RI between term and preterm neonates was not apparent early in development (at D3 and D8), but showed a significantly higher resistivity only from D21 in preterm neonates (0.78 [0.70 0.85]) compared with term neonates (0.61 [0.57 0.64]) (*p < *0.01) (Figure 5(b)). This finding may be due to the greater variance of this measurement because the influence of the vessel diameter on the RI is not accounted for.

### The RI in small vessels increases in preterm neonates within the first weeks of life

The RI in small vessels in the ACA branches ROI between the day of acquisition in preterm neonates showed a tendency for the RI to increase with postnatal age until D21 (Figure 5(a), grey significance stars). At D21, the RI was 0.66 ± 0.09, higher than at D3 (0.59 ± 0.08, *p < *0.001). After D21, the RI might slightly decrease (*p < *0.05). We should emphasise that at TEA, the preterm neonates had different postnatal ages, which probably increased the variance of this measurement.

### The slope of the RI versus diameter differs in preterm neonates at birth and tends to normalise over time

In the ACA branches ROI, the slope of the RI versus diameter tended to be lower for preterm neonates than term neonates at D3 (not significant) (Figure 5(c)). In preterm neonates, the slope increased with postnatal age (D3 < TEA [*p < *0.001], D8 < TEA [*p < *0.001] and D21 < TEA [*p < *0.001]) so that by TEA, the slope was almost the same as the control group of term neonates. Overall, the evolution of the RI along the vascular tree seems to be different just after birth for preterm neonates because of a higher RI in small vessels, as explained above. This effect is concealed when considering only the large vessels, as is done in the clinical practice, because the ACA branches RI values for the large vessels are very similar for preterm neonates at D3 (Figure 5(c), right end part of yellow curve) and term neonates (Figure 5(c), right end part of green curve) (no significant difference). With postnatal age, the resistivity also increases in larger vessels, which results in a term-like slope at TEA but with overall higher values of resistivity in preterm neonates.

### The RI developmental change of a vessel depends on the brain area and the vascular tree to which it belongs

We compared the RI developmental change observed in the ACA branches and the thalamic arteries by examining the slope of the RI versus diameter regression. The slope of the ACA branches (0.08 ± 0.07 mm^−1^) was significantly higher than the slope of the thalamic arteries (0.03 ± 0.08 mm^−1^, *p < *0.001; Figure 5(c) and (d)).

Of note, in Figure 5(c) and (d) the average resistivity for vessels of a given diameter is lower in the thalamic arteries than in the ACA branches. Quantitatively, for small vessels of the same 0.2 mm diameter, we found a lower RI in the thalamic arteries (0.43 [0.39 0.49]) than the ACA branches (0.58 [0.52 0.65]) (*p < *0.001). This type of comparison of the RI in vessels of the same diameter in different vascular domains was not previously possible. It shows that a clinical observation (a lower RI in the thalamic arteries than in the ACA branches) classically explained with presupposed vessel diameter considerations (thalamic arteries are smaller than ACA branches^25^) is not fully based on evidence. Indeed, because previous studies did not quantify the vessel diameter, the effect from the diameter and vascular region could not be differentiated. We have shown here that the RI in a vessel of a given diameter also depends on the vascular tree to which it belongs.

### The resistivity versus diameter slope is predictive of white matter lesions detected on MRI

**Figure 6.**
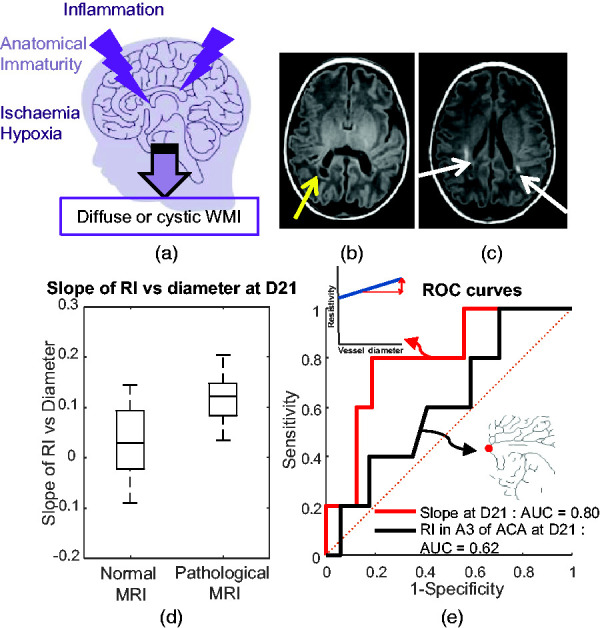
A potential new ultrasound biomarker to predict white matter injury (WMI). (a) Schematic representation of the causes of WMI, including inflammation, anatomical immaturity and ischaemia/hypoxia. (b) MRI image acquired at term-equivalent age (TEA) with abnormalities of the signal in the white matter: the yellow arrow indicates a cystic lesion located in the periventricular white matter. (c) MRI image acquired at TEA: the white arrows indicate linear and punctiform T1 hypersignals in the periventricular white matter. (d) Repartition of slope values of the resistivity index (RI) as a function of the diameter for neonates at postnatal day 21 (D21) who underwent MRI at TEA according to the normal and pathological groups. They are classified as pathological or normal based on analysis of the MRI images and (e) Receiver operating characteristic curve analysis for the slope at D21 (in red: area under the curve [AUC] of 0.80 and maximum Youden score of 80% sensitivity and 81% specificity) and the RI in A3 segment of anterior cerebral artery (ACA) at D21 (in black: AUC of 0.62 and maximum Youden score of 100% sensitivity and 29% specificity).

White matter injuries (WMI) have multiple causes, including neuro-inflammation and poor regulation of cerebral blood flow (Figure 6(a)). Thus, the two parameters – the slope and the RI in small vessels – might be relevant for predicting the occurrence of WMI. A subgroup of very preterm neonates (n = 24) underwent MRI at TEA, among whom 6 had detectable white matter lesions (see examples in Figure 6(b) and (c)). They were located either in the frontal or parieto-occipital white matter and either the left or right hemisphere.

When comparing slope and RI in small vessels for each day of acquisition, we noted a significantly higher mean slope in pathological preterm neonates at D21 (Figure 6(d); *p < *0.05). To assess the predictive value of the slope at D21, we generated a receiver operating characteristic (ROC) curve and calculated the area under the curve (AUC); it was 0.80 (Figure 6(e)). We chose the Youden index as the best delimitation criterion; it gave a sensitivity of 80% and specificity of 81%. For comparison, if we tried to classify neonates with an abnormal MRI based on the RI in the A3 segment of the ACA, we obtained a ROC curve with a much worse performance (AUC = 0.62; Figure 6(e)).

## Discussion

We have shown that UfD can dramatically change the clinical value of ultrasound-based measurements such as the RI. Their current poor predictive value might be linked to the lack of control of the conditions in which these measurements are performed in clinical practice. In particular, despite the standardisation of the measurement loci for radiologists (e.g. in the A3 segment of the ACA), the current clinical RI measurement gives only a partial view of the vascular resistance in a vascular tree: it is mostly measured at the entry points (big vessels) and without considering the local anatomical geometry (vessel diameter). Here we have shown that by using UfD, we can both consider the local vessel diameter and obtain a global resistance assessment over vascular trees by aggregating RI measurements over the whole FOV and linearly modelling them versus the local vessel diameter. This approach allowed us to obtain quantitative measurements that confirm trends that have already been identified in preterm neonates, such as the increase in resistivity with postnatal age and higher resistivity,^26,27^ but in a more subtle way (in smaller vessels and as early as D3). It allowed us to finely characterise and explain different resistive behaviours in different vascular trees (e.g. ACA branches versus thalamic arteries), independently of local vessel diameters. Assessing the resistivity in the small vessels also revealed differences in RI at birth when comparing premature neonates with term neonates, even if this difference is generally concealed when considering a RI measurement in the big vessels. This might be due to differences in the maturation of the vascular system downstream combined with a different adaptability at birth to the outside world. As it is known that premature neonates have a higher risk of brain injuries after birth, further studies are needed to linked resistivity and brain vulnerability. Our study has several limitations to answer these questions. In our data we could not observe any significant differences in resistivity between preterm born between 24 and 28 weeks and those born between 28 and 32 weeks, who are usually considered differently from a clinical care point of view (see supplemental figure 2), but assessing this question might require larger cohorts and finer gestational age sampling. Sex difference was also not investigated due to the relatively small number of patients: it has been shown that it does not impact cerebral blood flow parameters such as RI measured in big vessels^28^, but it could be different in small vessels. Finally, and technical aspects about this are discussed below, a finer monitoring in time should be performed, both on the preterm patients and on the term controls, as the latter probably also undergo changes of cerebral resistivity during the days and weeks after birth, something that was not investigated here, mostly because those patients leave early the hospital.

At last, we have shown in a small cohort of preterm neonates that a new parameter (slope of the linear modelling) of this global resistivity assessment has a good predictive value to detect white matter lesions compared with classical RI measurement. We could better classify neonates with abnormal MRI findings by using the slope parameter, probably because it considers information of the vascular resistance of the whole vascular tree, compared with partial information obtained through the typical one measurement at one location (the RI in the A3 segment of the ACA) obtained in the clinics. This example shows that new information obtained through a global RI analysis over a wide FOV, such as the slope and RI in small vessels (<0.2 mm diameter), might offer valuable clinical biomarkers to detect earlier the risk of WMI. It paves the way for new applications in diseases related to cerebrovascular dysfunction.

Monitoring the RI while carefully controlling the vessel location and anatomy where the measurement is made have been identified as crucial factors by clinicians.^25^ Even if the probe is held manually by a trained operator, thanks to UfD we can eliminate the difficulty of placing the ultrasound probe in the exact same location to compare values because we can simultaneously assess the RI in each FOV. This approach removes some variability due to the insonification angle. Moreover, from these measures we could extract two global parameters that consider the regional localisation on the vascular tree and the size of the vessels.

Another crucial need identified long ago by specialists of perinatal pathologies and brain injuries of premature infants is quantitative real-time monitoring of the cerebral circulation.^29^ NeoDoppler^30^ is an attempt to monitor the cerebral circulation continuously at different depths, but the loss of spatial information seems to be a major drawback in light of our results showing how the local vessel diameter and the anatomical location of the vessel change the RI. Here, our measurements were only four snapshots at given times, which we defined early in the protocol design. However, continuous monitoring would help to investigate more precisely the evolution of resistivity with postnatal age. Studies^27,31^ have shown that the RI is inversely correlated with postnatal time within a much earlier period of time (a hours to a few days) than the one we studied. It is hard to compare studies as the measurement timeline can be very different, but it may hint at very complex RI variation patterns during the postnatal period. Our method has all the necessary elements to move to continuous monitoring in the future, an approach that could help to detect abnormalities earlier.

With a spatial resolution of 0.2 mm, UfD provides the highest resolution for bedside imaging of the neonatal cerebral vasculature while maintaining good sensitivity in small vessels. However, it might be interesting to obtain information in even smaller vessels such as arterioles or capillaries, which have a main role in regulating cerebral perfusion.^32^ Critically, they are the first vessels to be impacted by preterm birth due to their immaturity at this stage. Increasing the ultrasound frequency could improve the spatial resolution, but at the cost of a lower penetration depth and sensitivity. Researchers have shown that both peak-systolic velocity (PSV) and end-diastolic velocity (EDV) increase with gestational age and postnatal age for preterm infants and that this is poorly reflected in the RI being the ratio between PSV and EDV.^33,34^ We tried to look directly at PSV and EDV while correcting for the in-plane angulation of the vessel, but the measured values had high variance, potentially due to the uncorrectable out-of-plane vessel angle, and we preferred to keep the RI measurement, which has the advantage of normalising these angulation effects.

To conclude, we could extract from our unsupervised analysis two new global resistivity markers that provide a reliable measurement, without mixing spatial information. These parameters might be early predictive markers of WMI in preterm neonates as assessed on MRI at TEA in our cohort. Using UfD for continuous monitoring may further improve the detection of cerebrovascular dysfunction and treatment follow-up.

## Supplemental Material

sj-pdf-1-jcb-10.1177_0271678X241232197 - Supplemental material for Quantification of brain-wide vascular resistivity via ultrafast Doppler in human neonates helps early detection of white matter injury Supplemental material, sj-pdf-1-jcb-10.1177_0271678X241232197 for Quantification of brain-wide vascular resistivity via ultrafast Doppler in human neonates helps early detection of white matter injury by Flora Faure, Jérôme Baranger, Marianne Alison, Béatrice Boutillier, Alice Frérot, Chung Lim, Grégory Planchette, Mickael Prigent, Mickaël Tanter, Olivier Baud, Valérie Biran and Charlie Demené in Journal of Cerebral Blood Flow & Metabolism

sj-pdf-2-jcb-10.1177_0271678X241232197 - Supplemental material for Quantification of brain-wide vascular resistivity via ultrafast Doppler in human neonates helps early detection of white matter injury Supplemental material, sj-pdf-2-jcb-10.1177_0271678X241232197 for Quantification of brain-wide vascular resistivity via ultrafast Doppler in human neonates helps early detection of white matter injury by Flora Faure, Jérôme Baranger, Marianne Alison, Béatrice Boutillier, Alice Frérot, Chung Lim, Grégory Planchette, Mickael Prigent, Mickaël Tanter, Olivier Baud, Valérie Biran and Charlie Demené in Journal of Cerebral Blood Flow & Metabolism

sj-pdf-3-jcb-10.1177_0271678X241232197 - Supplemental material for Quantification of brain-wide vascular resistivity via ultrafast Doppler in human neonates helps early detection of white matter injury Supplemental material, sj-pdf-3-jcb-10.1177_0271678X241232197 for Quantification of brain-wide vascular resistivity via ultrafast Doppler in human neonates helps early detection of white matter injury by Flora Faure, Jérôme Baranger, Marianne Alison, Béatrice Boutillier, Alice Frérot, Chung Lim, Grégory Planchette, Mickael Prigent, Mickaël Tanter, Olivier Baud, Valérie Biran and Charlie Demené in Journal of Cerebral Blood Flow & Metabolism

## References

[1] Volpe JJ. Brain injury in premature infants: a complex amalgam of destructive and developmental disturbances. Lancet Neurol 2009; 8: 110–124. 19081519 10.1016/S1474-4422(08)70294-1 PMC2707149

[2] Ancel PY Goffinet F Kuhn P , et al. Survival and morbidity of preterm children born at 22 through 34 weeks’ gestation in France in 2011 results of the EPIPAGE-2 cohort study. JAMA Pediatr 2015; 169: 230–238. 25621457 10.1001/jamapediatrics.2014.3351

[3] Volpe JJ. Neurobiology of periventricular leukomalacia in the premature infant. Pediatr Res 2001; 50: 553–562. 11641446 10.1203/00006450-200111000-00003

[4] Guo T Duerden EG Adams E , et al. Quantitative assessment of white matter injury in preterm neonates. Neurology 2017; 88: 614–622. 28100727 10.1212/WNL.0000000000003606 PMC5317385

[5] Dempsey EM Barrington KJ Marlow N , et al. Management of hypotension in preterm infants (the HIP trial): a randomised controlled trial of hypotension management in extremely low gestational age newborns. Neonatology 2014; 105: 275–281. 24576799 10.1159/000357553

[6] Couture A Veyrac C. Transfontanellar Doppler imaging in neonates. Berlin, Heidelberg: Springer, 2001. 10.1007/s00330-001-1150-z 11734933

[7] Brew N Walker D Wong FY. Cerebral vascular regulation and brain injury in preterm infants. Am J Physiol Regul Integr Comp Physiol 2014; 306: R773–R786. 24647591 10.1152/ajpregu.00487.2013

[8] Baranger J Villemain O Wagner M , et al. Brain perfusion imaging in neonates. NeuroImage Clin 2021; 31: 102756. 34298475 10.1016/j.nicl.2021.102756 PMC8319803

[9] Da Costa CS Greisen G Austin T. Is near-infrared spectroscopy clinically useful in the preterm infant? Arch Dis Child Fetal Neonatal Ed 2015; 100: F558–F561. 26215405 10.1136/archdischild-2014-307919

[10] Veyrac C Couture A Saguintaah M , et al. Brain ultrasonography in the premature infant. Pediatr Radiol 2006; 36: 626–635. 16770667 10.1007/s00247-006-0202-6

[11] Montaldo G Tanter M Bercoff J , et al. Coherent plane-wave compounding for very high frame rate ultrasonography and transient elastography. IEEE Trans Ultrason Ferroelectr Freq Control 2009; 56: 489–506. 19411209 10.1109/TUFFC.2009.1067

[12] Mace E Montaldo G Osmanski BF , et al. Functional ultrasound imaging of the brain: Theory and basic principles. IEEE Trans Ultrason Ferroelectr Freq Control 2013; 60: 492–506. 23475916 10.1109/TUFFC.2013.2592

[13] Bercoff J Montaldo G Loupas T , et al. Ultrafast compound doppler imaging: providing full blood flow characterization. IEEE Trans Ultrason Ferroelectr Freq Control 2011; 58: 134–147. 21244981 10.1109/TUFFC.2011.1780

[14] Demené C Pernot M Biran V , et al. Ultrafast doppler reveals the mapping of cerebral vascular resistivity in neonates. J Cereb Blood Flow Metab 2014; 34: 1009–1017. 24667916 10.1038/jcbfm.2014.49 PMC4050246

[15] Peeples ES Mehic E Mourad PD , et al. Fast Doppler as a novel bedside measure of cerebral perfusion in preterm infants. Pediatr Res 2016; 79: 333–338. 26539662 10.1038/pr.2015.227

[16] Demené C Maresca D Kohlhauer M , et al. Multi-parametric functional ultrasound imaging of cerebral hemodynamics in a cardiopulmonary resuscitation model. Sci Rep 2018; 8: 16436–10. 30401816 10.1038/s41598-018-34307-9 PMC6219610

[17] Kim HG Lee JH. Feasibility of ultrafast doppler technique for cranial ultrasound in neonates. Med Ultrason 2019; 21: 288–293. 31476209 10.11152/mu-1901

[18] Demené C Deffieux T Pernot M , et al. Spatiotemporal clutter filtering of ultrafast ultrasound data highly increases Doppler and fUltrasound sensitivity. IEEE Trans Med Imaging 2015; 34: 2271–2285. 25955583 10.1109/TMI.2015.2428634

[19] Baranger J Arnal B Perren F , et al. Adaptive spatiotemporal SVD clutter filtering for ultrafast Doppler imaging using similarity of spatial singular vectors. IEEE Trans Med Imaging 2018; 37: 1574–1586. 29969408 10.1109/TMI.2018.2789499

[20] Prabhu P Duraiswamy K. Enhanced dark block extraction method performed automatically to determine the number of clusters in unlabeled data sets. Int J Comput Commun Control 2013; 8: 275–293.

[21] Deffieux T Demené C Tanter M. Functional ultrasound imaging: a new imaging modality for neuroscience. Neuroscience 2021; 474: 110–121. 33727073 10.1016/j.neuroscience.2021.03.005

[22] Jerman T Pernus F Likar B , et al. Enhancement of vascular structures in 3D and 2D angiographic images. IEEE Trans Med Imaging 2016; 35: 2107–2118. 27076353 10.1109/TMI.2016.2550102

[23] Bonnefous O Pesqué P. Time domain formulation of pulse-Doppler ultrasound and blood velocity estimation by cross correlation. Ultrason Imaging 1986; 8: 73–85. 2946098 10.1177/016173468600800201

[24] Osmanski B-F Bercoff J Montaldo G , et al. Cancellation of doppler intrinsic spectral broadening using ultrafast doppler imaging. IEEE Trans Ultrason, Ferroelect, Freq Contr 2014; 61: 1396–1408.

[25] Ecury-Goossen GM Raets MMA Camfferman FA , et al. Resistive indices of cerebral arteries in very preterm infants: values throughout stay in the neonatal intensive care unit and impact of patent ductus arteriosus. Pediatr Radiol 2016; 46: 1291–1300. 27259991 10.1007/s00247-016-3615-x PMC4943974

[26] Zamora C Tekes A Alqahtani E , et al. Variability of resistive indices in the anterior cerebral artery during fontanel compression in preterm and term neonates measured by transcranial duplex sonography. J Perinatol 2014; 34: 306–310. 24526007 10.1038/jp.2014.11

[27] Seibert JJ McCowan TC Chadduck WM , et al. Duplex pulsed doppler US versus intracranial pressure in the neonate: clinical and experimental studies. Radiology 1989; 171: 155–159. 2648468 10.1148/radiology.171.1.2648468

[28] Aranha CA Lederman HM Segre CAM. Color doppler evaluation of the influence of type of delivery, sex, postnatal age and time post feeding on full term healthy newborns cerebral blood flow. Arq Neuropsiquiatr 2009; 67: 463–473. 19623445 10.1590/s0004-282x2009000300017

[29] Volpe JJ. Brain injury in the premature infant: neuropathology, clinical aspects, pathogenesis, and prevention. Clin Perinatol 1997; 24: 567–587. 9394861

[30] Vik SD Torp H Follestad T , et al. NeoDoppler: new ultrasound technology for continuous cerebral circulation monitoring in neonates. Pediatr Res 2020; 87: 95–103. 31404920 10.1038/s41390-019-0535-0 PMC6960092

[31] Forster DE Koumoundouros E Saxton V , et al. Cerebral blood flow velocities and cerebrovascular resistance in normal‐term neonates in the first 72 hours. J Paediatr Child Health 2017; 54: 61–68. 28845537 10.1111/jpc.13663

[32] Grubb S Cai C Hald BO , et al. Precapillary sphincters maintain perfusion in the cerebral cortex. Nat Commun 2020; 11: 395. 31959752 10.1038/s41467-020-14330-z PMC6971292

[33] Deeg KH Rupprecht T. Pulsed doppler sonographic measurement of normal values for the flow velocities in the intracranial arteries of healthy newborns. Pediatr Radiol 1989; 19: 71–78. 2646587 10.1007/BF02387890

[34] Romagnoli C Giannantonio C De Carolis MP , et al. Neonatal color doppler US study: normal values of cerebral blood flow velocities in preterm infants in the first month of life. Ultrasound Med Biol 2006; 32: 321–331. 16530090 10.1016/j.ultrasmedbio.2005.12.007

