# Supplementary material for: Quantification of brain-wide vascular resistivity via ultrafast Doppler in human neonates helps early detection of white matter injury
Source: J Cereb Blood Flow Metab. 2024 Feb 10:0271678X241232197. Online ahead of print. doi: 10.1177/0271678X241232197 (PMC11639668; doi:10.1177/0271678X241232197)
Supplement: sj-pdf-1-jcb-10.1177_0271678X241232197 - Supplemental material for Quantification of brain-wide vascular resistivity via ultrafast Doppler in human neonates helps early detection of white matter injury [file sj-pdf-1-jcb-10.1177_0271678X241232197.pdf]

Supplemental Figure 1

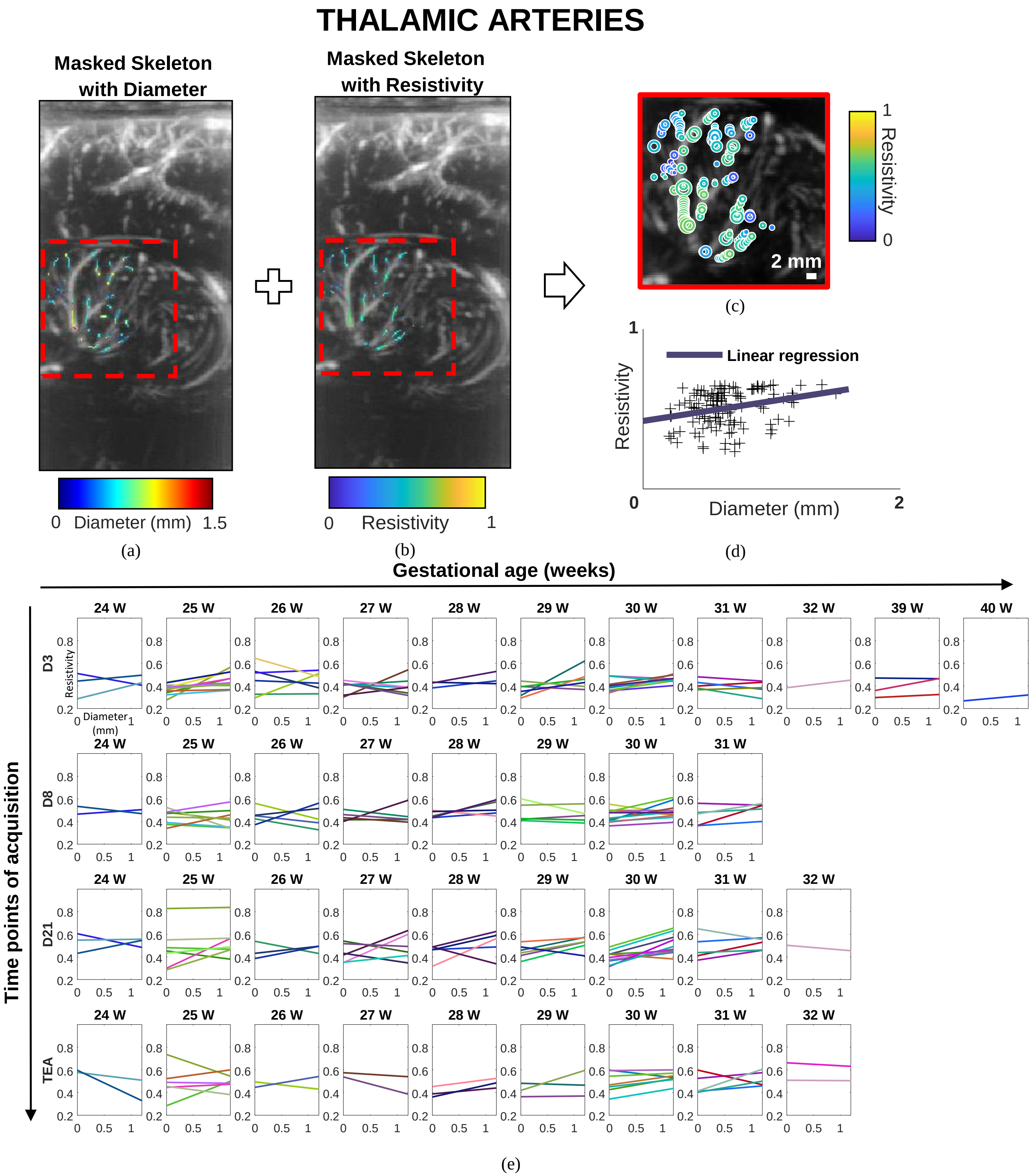

Supplemental Figure 1. Resistivity versus artery diameter analysis on the defined thalamic region of interest (ROI, red box). (a) Doppler image (in greyscale) with the local diameter (in the given colour scale) superimposed in the thalamic ROI. (b) Doppler image (in greyscale) with the local resistivity (in the given colour scale) superimposed in the thalamic ROI. (c) Coincident mapping of resistivity and diameter (the size of the circles is proportional to the diameter and the colours represent resistivity according to the colour map). (d) The RI and diameters can also be gathered in a resistivity versus diameter plot (black crosses), before performing linear regression of resistivity as a function of diameter (purple line). (e) RI versus diameter linear regression for all patients grouped by gestational age at birth (in weeks [W]) and day of acquisition (postnatal day 3 [D3], D8, D21 and term-equivalent age [TEA]) in the thalamus. Each patient is represented by a specific colour.
