# Supplementary material for: Quantification of brain-wide vascular resistivity via ultrafast Doppler in human neonates helps early detection of white matter injury
Source: J Cereb Blood Flow Metab. 2024 Feb 10:0271678X241232197. Online ahead of print. doi: 10.1177/0271678X241232197 (PMC11639668; doi:10.1177/0271678X241232197)
Supplement: sj-pdf-2-jcb-10.1177_0271678X241232197 - Supplemental material for Quantification of brain-wide vascular resistivity via ultrafast Doppler in human neonates helps early detection of white matter injury [file sj-pdf-2-jcb-10.1177_0271678X241232197.pdf]

Supplemental Figure 2

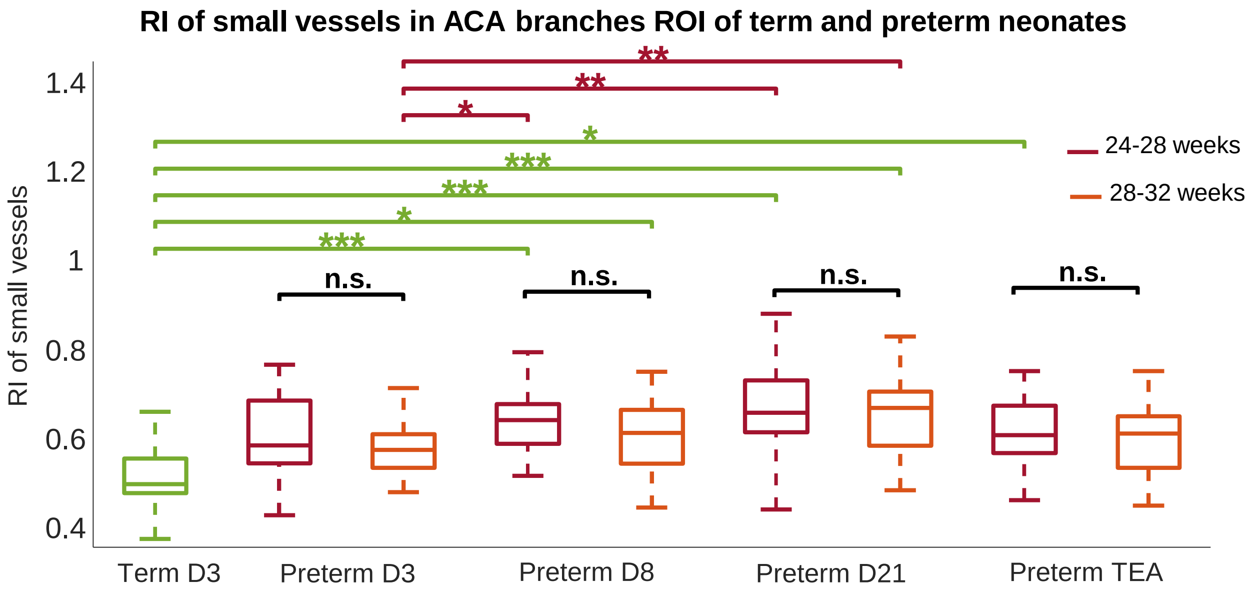

Supplemental Figure 2. Comparison of resistivity for term neonates and two groups of preterm neonates (grouped by gestational age at birth) according to postnatal days. RI in small vessels in branches of the anterior cerebral artery (ACA) evaluated by the value of regression for a diameter of 0.2 mm grouped by day of acquisition (postnatal day 3 [D3], D8, D21 and term-equivalent age [TEA]) and the gestational age group (term neonates in green, preterm neonates born between 24 weeks [W] and 28 W in red and preterm neonates born between 28 W and 32 W in orange). One-way analysis of variance was performed followed by post hoc Bonferroni correction for multiple comparisons (n.s. = not significant; * $p < 0.05$, ** $p < 0.01$, *** $p < 0.001$).
