# Supplementary material for: Quantification of brain-wide vascular resistivity via ultrafast Doppler in human neonates helps early detection of white matter injury
Source: J Cereb Blood Flow Metab. 2024 Feb 10:0271678X241232197. Online ahead of print. doi: 10.1177/0271678X241232197 (PMC11639668; doi:10.1177/0271678X241232197)
Supplement: sj-pdf-3-jcb-10.1177_0271678X241232197 - Supplemental material for Quantification of brain-wide vascular resistivity via ultrafast Doppler in human neonates helps early detection of white matter injury [file sj-pdf-3-jcb-10.1177_0271678X241232197.pdf]

Supplemental Table 1: Characteristics of the patients and their prenatal history

TABLE I
CHARACTERISTICS OF THE PATIENTS AND THEIR PRENATAL HISTORY

	PRETERM (N = 74)	TERM (N = 10)
Gestational age at birth (weeks), mean ± standard deviation	28.5 ± 2.2	40.1 ± 0.8
Birthweight (g), mean ± standard deviation	1080 ± 318	3413 ± 657
Male/female sex, n (%)	35/39 (47/53%)	5/5 (50/50%)
Antenatal steroids, n (%)	69 (93%)	0 (0%)
Multiple pregnancy, n (%)	30 (41%)	0 (0%)
Chorioamnionitis, n (%)	15 (20%)	0 (0%)
Caesarean section, n (%)	31 (42%)	2 (20%)
Apgar score at 1 minute, median (interquartile range)	7 (4–9)	10 (7–10)
Apgar score at 5 minutes median (interquartile range)	9 (7–10)	10 (10–10)
